# Efficiency of Ozone Applied in Flow and at Low Pressures in the Inactivation of *Salmonella* in Black Peppercorns (*Piper nigrum* L.) and the Effects of Ozone Treatment on Grain Quality and Essential Oil Composition

**DOI:** 10.3390/foods14132215

**Published:** 2025-06-24

**Authors:** Handina da Graça Lurdes Langa Massango, Lêda Rita D’Antonino Faroni, Maria Cristina Dantas Vanetti, Ernandes Rodrigues de Alencar, Marcus Vinícius de Assis Silva, Alessandra Aparecida Zinato Rodrigues, Paulo Roberto Cecon, Carollayne Gonçalves Magalhães, Daniele Almeida Teixeira, Letícia Elisa Rossi

**Affiliations:** 1 Department of Agricultural Engineering, Universidade Federal de Viçosa, Viçosa 36570-900, MG, Brazil; handina.langa@ufv.br (H.d.G.L.L.M.); ernandes.alencar@ufv.br (E.R.d.A.); marcus.assis@ufv.br (M.V.d.A.S.); alessandra.rodrigues@ufv.br (A.A.Z.R.); carollayne.magalhaes@ufv.br (C.G.M.); daniele.almeida@ufv.br (D.A.T.); leticia.rossi@ufv.br (L.E.R.); 2 Department of Microbiology, Universidade Federal de Viçosa, Viçosa 36570-900, MG, Brazil; mvanetti@ufv.br; 3 Department of Statistics, Universidade Federal de Viçosa, Viçosa 36570-900, MG, Brazil; cecon@ufv.br

**Keywords:** ozonation, *Piper nigrum* L., *Salmonella*, bacterial inactivation, essential oils

## Abstract

Food contamination by *Salmonella* poses a significant public health risk, rendering products unfit for consumption. This study aimed to evaluate the efficiency of ozone gas (O_3_), applied in flow and at low pressures, in inactivating *Salmonella* on black peppercorns (*Piper nigrum* L.). Samples were inoculated with a cocktail of four *Salmonella* serotypes and subjected to ozonation under flow or low-pressure conditions in a hypobaric chamber. For the flow treatment, ozone gas at 16 mg L^−1^ was humidified by passing it through a 40% (*w*/*v*) sodium chloride solution and applied for 2, 4, and 8 h. For the hypobaric chamber treatment, an inlet O_3_ concentration of 60 mg L^−1^ was used, with 10, 15, and 20 injections. The results showed that, under flow ozonation for 8 h, *Salmonella* was absent in 25 g of the sample. Ozone treatment increased pH, total titratable acidity (TTA), antioxidant activity (DPPH*), lightness (L*), color saturation (C*), total phenolic content (TPC), and the concentration of major essential oil compounds in all treatments. Under low-pressure ozonation, *Salmonella* persisted in all tested conditions, along with changes in color difference (∆E*), moisture content, TTA, DPPH*, L*, C*, pH, TPC, and the concentration of major essential oil compounds. The essential oil yield was not affected. Although the application of ozone at low pressures reduced *Salmonella* contamination, it was not sufficient for complete inactivation under the tested conditions. However, the flow-applied ozone treatment proved effective in the inactivation of *Salmonella* in black peppercorns.

## 1. Introduction

Aromatic herbs and spices have been an essential part of human nutrition since early human history. They have been used for thousands of years to enhance the flavor, color, and aroma of food and are also recognized for their preservative and medicinal properties [1,2]. Black pepper (*Piper nigrum* L.), also known as “the king of spices,” is the most economically important condiment among the spices traded worldwide [3]. Global black pepper production fluctuates throughout the year according to the harvest seasons of the main producing countries [4]. Brazil is the world’s second-largest producer and exporter of black pepper, behind only Vietnam [5].

Black pepper is primarily used in food preparation, but it is also employed in the pharmaceutical industry for the production of cosmetics and perfumes [3] and as a natural insecticide [6]. However, its use in food preparation can be a source of pathogen contamination, potentially leading to health issues for consumers [7]. Contamination may occur due to improper handling during harvesting, post-harvesting, and product processing, directly affecting the quality and shelf life of foods to which this spice is added [8]. During the post-harvest stage, black peppercorns are also vulnerable to contamination during drying, making them highly susceptible to the presence of dust, animal feces, and microorganisms [8,9,10].

Bacteria of the genus *Salmonella* pose a threat to the black pepper production chain, and once contamination occurs, they can survive for extended periods under low water activity conditions [11,12]. As water activity decreases, these bacteria exhibit increased thermal resistance, making inactivation more difficult [13]. The *Salmonella* genus comprises numerous serotypes and is among the main pathogens responsible for foodborne bacterial diseases. Most of these serotypes are pathogenic to humans, and symptom severity can vary depending on differences in pathogenic mechanisms, as well as factors such as host age and immune response [14,15].

For microbial decontamination of spices whose active compounds are sensitive to high temperatures, gamma irradiation has been used, resulting in products that are safe for consumption and of improved quality [16]. However, despite being regulated in many countries, irradiation is still not widely accepted by consumers due to concerns about potential reductions in the nutritional composition of certain foods [17,18,19]. As an alternative, scientific investigations have been conducted to explore innovative treatments for the microbial decontamination of spices, with ozone gas being one of the proposed options [20].

Ozone (O_3_) is a powerful oxidizing agent naturally present in the environment [21,22]. Even when applied at low concentrations, it is known to inhibit the growth of bacteria, filamentous fungi, yeasts, parasites, and viruses [23,24]. However, ongoing research aims to improve application technology [25]. Ozone has a half-life of 20 to 50 min at atmospheric pressure [26], and for this reason, its application to grains is typically carried out through forced air movement [25,27,28,29].

Another strategy for applying ozone gas to control microorganisms is its use under low-pressure conditions [30]. Positive effects of this approach have already been studied in popcorn (*Zea mays everta*) and common beans (*Phaseolus vulgaris*) for the control of insects and fungi [30,31]. Therefore, it is expected that combining ozone application with a low-pressure system may enhance its stability and increase its effectiveness in controlling bacteria such as *Salmonella*, especially in packaged products. However, the effects of low-pressure ozone application on black pepper have not yet been investigated.

For the use of O_3_ in microbiological decontamination, whether through flow systems or low-pressure injection systems, its effects on grain quality must be well understood. Ozone exposure has been reported to influence the chemical composition and bioactive properties of essential oils in plants, sometimes enhancing the concentration of medicinal compounds and antioxidants, while in other cases inducing oxidative degradation of sensitive volatile compounds [32,33]. Furthermore, ozone has demonstrated efficacy in microbial decontamination of medicinal plants without significantly altering essential oil yields [34]. These contrasting effects highlight the importance of evaluating ozone treatment parameters to optimize both microbial safety and product quality.

Therefore, this study aimed to evaluate the efficiency of ozone gas, applied in flow and under low-pressure conditions, in the decontamination of black peppercorns contaminated with *Salmonella*, as well as to investigate its impact on product quality, particularly regarding the characteristics of the essential oil.

## 2. Materials and Methods

### 2.1. Raw Material Procurement and Initial Characterization

The black pepper used in this study was purchased from Temperart Indústria e Comércio de Produtos Alimentícios LTDA, located in São Paulo, Brazil. Prior to ozonation, samples were taken for the initial characterization of the grains, including quality analyses. The samples were stored in woven polypropylene bags at room temperature (approximately 25 °C) until the start of the experiments, without compromising grain quality. All experiments were carried out using black pepper samples from the same batch.

### 2.2. Preparation of Inoculated Black Pepper Samples

#### 2.2.1. Microbial Cultures and Growth Conditions

A *Salmonella* cocktail composed of four *S. enterica* strains (*Salmonella* Enteritidis PT4, *S.* Enteritidis ATCC 13076, *Salmonella* Typhimurium ATCC 14028, and *Salmonella* Choleraesuis) was prepared and used to inoculate the black pepper samples. The activation of the cultures was carried out from stock cultures of each strain stored at −80 °C. A volume of 100 μL of bacterial cells from each strain was inoculated into 3 mL of tryptic soy broth (TSB) and incubated at 35 °C for 24 h.

#### 2.2.2. Inoculum Preparation and Black Pepper Inoculation

For the inoculation of black pepper, aliquots of 1 mL and 2.5 mL of each of the four active cultures were added to 10 g and 25 g of black pepper samples, respectively, for quantitative and qualitative tests. The inoculum was uniformly distributed over the samples, which were placed in organza sachets. The final concentration of the inoculum was adjusted to approximately 10^6^ CFU g^−1^ of black pepper. To confirm the bacterial population, samples of black pepper previously inoculated with the *Salmonella* cocktail were plated on Salmonella–Shigella (SS) agar and incubated at 35 °C for 48 h.

### 2.3. Ozone Gas Treatment in a Flow System

A cylindrical column made of polyvinyl chloride (PVC), 0.5 m in length and 0.15 m in diameter, was constructed for the ozonation of black pepper samples in a flow system. A metal screen was installed at the base of the column, positioned 0.10 m above the bottom to form a plenum chamber, which supported the pepper grains and allowed for the uniform distribution of ozone gas throughout the column.

**Figure 1:**
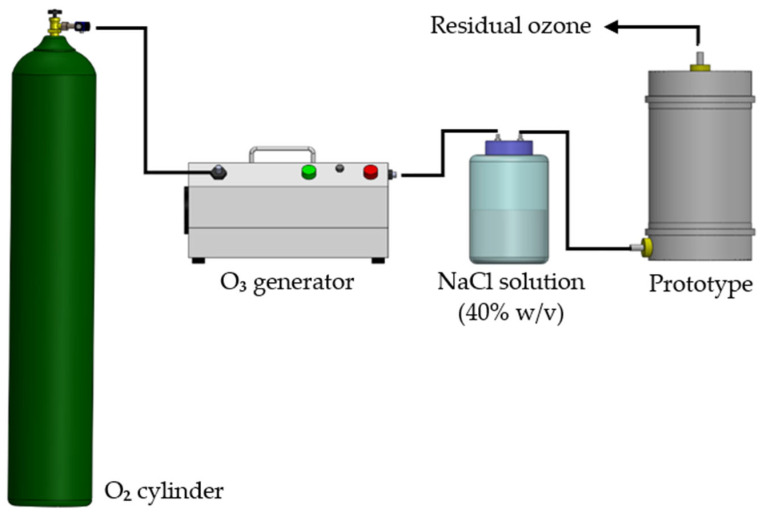
Schematic representation of the experimental procedure for ozone application in a flow system.

Inlet and outlet ports for ozone gas were installed at the bottom and top of the cylindrical column. Samples of 1.5 kg of black peppercorns were exposed to O_3_ gas at a concentration of 16 mg L^−1^ and a flow rate of 0.5 L min^−1^ for 2, 4, and 8 h. To adjust and control the relative humidity, the ozone gas was humidified by passing through a saturated sodium chloride solution (40% *w*/*v*). The ideal experimental conditions were determined based on preliminary tests. Figure 1 illustrates the experimental setup for the ozone application in a flow system.

Ozone was generated using the corona discharge technique with a portable generator, model 0&L 3.0 RM (Ozone & Life, São José dos Campos, SP, Brazil), supplied by an industrial oxygen cylinder (99.9% purity). The generated ozone was quantified using the iodometric method, as described by Rakness et al. [35].

The experiment followed a completely randomized design (CRD), with three treatments (2, 4, and 8 h) and three replicates. Each replicate consisted of three organza sachets containing 10 g of black pepper inoculated with *Salmonella*, used for the quantitative test. For the qualitative test, sachets containing 25 g of *Salmonella*-inoculated samples were used. The organza sachets containing the inoculated samples were placed in the middle of the grain mass. For the control treatment, black pepper was inoculated with *Salmonella* and kept under ambient conditions for 2, 4, and 8 h, followed by microbiological analyses.

### 2.4. Ozone Gas Application in a Closed Low-Pressure System

**Figure 2:**
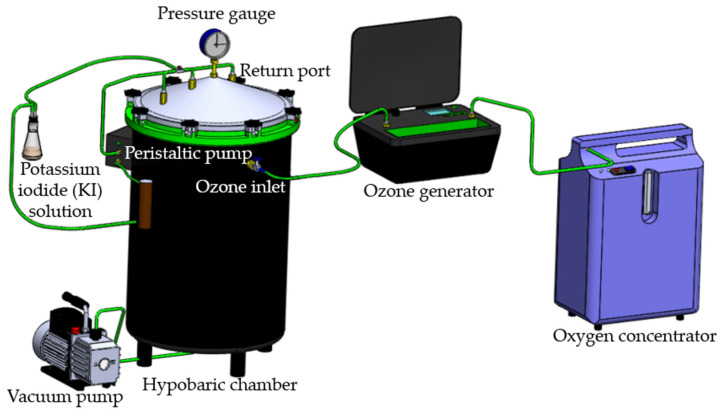
Schematic representation of the experimental procedure for ozone application under low-pressure conditions.

Ozone was generated using an industrial ozone generator, model M10i (myOZONE M10i, Jaguariúna, SP, Brazil). The gas was produced from oxygen supplied by an oxygen concentrator, model Mark 5 Plus (NIDEK Medical, Birmingham, AL, USA). For the ozonation of black peppercorns, a hypobaric chamber, model CV10 (myOZONE CV10, Jaguariúna, SP, Brazil), with a volumetric capacity of 70 L was used (Figure 2).

The internal pressure of the chamber was reduced to 350 hPa using a vacuum pump, model VP 260ND (TIPI—Professional Refrigeration, Caxias, RS, Brazil). Ozone gas was injected into the chamber at a volumetric flow rate of 1.0 L min^−1^ until a pressure of 1000 hPa was reached, following the methodology described by Silva et al. [30]. An input concentration of 60 mg L^−1^ was used, and the experiment followed a completely randomized design (CRD), with three treatments (10, 15, and 20 gas injections) and three replicates. The injections were performed at regular intervals of 47 min, which was the time required for the internal pressure of the hypobaric chamber to reach 1000 hPa. For the control treatment, contaminated black pepper was kept under ambient conditions.

Preliminary tests were carried out to determine the half-life and the time required for ozone to reach a pressure of 1000 hPa. The reaction time of ozone within the grain mass was 38 min, which corresponded to the ozone half-life in black pepper. For the ozone treatment in the hypobaric chamber, a mass of 3.5 kg of black peppercorns was placed in braided polypropylene bags (0.15 m × 0.50 m). Organza sachets containing 10 g and 25 g of black pepper inoculated with a *Salmonella* cocktail were distributed throughout the grain mass at the bottom, middle, and top of the packaging.

### 2.5. Microbiological Analyses

#### 2.5.1. Quantitative Test for Salmonella Analysis

Samples of 10 g of black pepper, either exposed or not exposed to ozone gas and previously inoculated with the *Salmonella* cocktail, were subjected to serial dilutions in 90 mL of sterile peptone saline solution (0.1% peptone, *w*/*v*, 0.85% NaCl, *w*/*v*), enriched with 2% sterile-filtered corn oil [36]. Subsequently, 1 mL aliquots of each dilution were plated on SSA culture medium for *Salmonella* analysis, following the guidelines of the American Public Health Association (APHA) and the International Standards Organization (ISO) (standards 4833-1:2013 [37] and 21527-1/2:2008 [38]). The plating was performed using the pour plate method, in duplicate. Plates were incubated at 35 ± 2 °C for 48 h for colony counting and calculation of CFU g^−1^.

#### 2.5.2. Qualitative Test for Salmonella Analysis (Presence/Absence in 25 g of Sample)

To assess the presence or absence of *Salmonella* in black pepper treated with ozone under flow-based and low-pressure systems, 25 g of contaminated black pepper exposed to ozone gas was aseptically added to 225 mL of TSB. The samples were left at room temperature for 1 h, followed by incubation at 37 °C for 24 h.

For selective enrichment and isolation of *Salmonella*, 100 μL of the pre-enriched sample was transferred to 10 mL of Rappaport Vassiliadis (RV) broth and 1 mL of the same sample to 10 mL of tetrathionate (TT) broth, both incubated at 37 °C for 24 h. After incubation, aliquots were streaked onto xylose lysine deoxycholate (XLD) agar, Hektoen enteric agar, and SSA. Plates were incubated at 37 °C for 24 h and examined for the presence of typical *Salmonella* colonies

### 2.6. Analysis of Bioactive Compounds and Quality of Black Pepper

To evaluate the bioactive compounds and quality of black pepper, samples not contaminated with *Salmonella* were used. These samples were ozonated under flow for 2, 4, and 8 h and under low pressure for 10, 15, and 20 injections. For the control treatment, non-ozonated and non-contaminated black peppercorns were used.

#### 2.6.1. Preparation of Black Pepper Extracts for Bioactive Compound Analysis

For the preparation of the extracts, 1.0 g of each ground black pepper sample was weighed and labeled, followed by the addition of 20 mL of an extraction solution composed of methanol, water, and acetic acid in the proportions of 70, 30, and 5 *v*/*v*, respectively. The mixture was shaken in an orbital shaker (SL-222, Solab, Piracicaba, SP, Brazil) at 240 rpm for 20 min. Then, the mixture was centrifuged at 2000 rpm for 20 min at 25 °C using a tube centrifuge (model 0222TM2, QUIMIS Scientific Equipment LTDA, Diadema, SP, Brazil). The supernatant was collected in Falcon tubes for further analyses.

#### 2.6.2. Total Phenolic Contents (TPC)

The quantification of total phenolic contents (TPC) in the samples was performed using the Folin–Ciocalteu method, adapted as described by Singleton and Rossi [39]. A volume of 0.6 mL of each sample was added to 3 mL of previously diluted Folin–Ciocalteu reagent (1:5). After 3 min, 2.4 mL of 7.5% sodium carbonate (Na_2_CO_3_) was added, and the mixture was kept at rest for 60 min in the dark.

Absorbance readings were performed using a spectrophotometer (Femto-Cirrus 80 ST, Femto, São Paulo, SP, Brazil) at a wavelength of 760 nm. The reaction was carried out in sextuplicate. The standard curve was prepared using gallic acid (200 mg L^−1^). Based on the standard curve, the TPC was determined, and the results were expressed as mg of gallic acid per 100 g of sample. The calculation of TPC was performed according to Equation (1).

(1) $$TPC\;(mg\;gallic\;acid\;100\;g^{-1}\;of\;sample)=\frac{GAE\times 100}{Extract\;D.}$$

where:

TPC—total phenolic content

GAE—gallic acid equivalent (obtained from the standard curve)

Extract D.—dilution of the extract or sample (grams)

#### 2.6.3. Antioxidant Activity (DPPH*)

The antioxidant activity was determined via the free radical DPPH* (2,2-diphenyl-1-picrylhydrazyl) scavenging method, using the methodology adapted from Brand-Williams et al. [40]. A total of 150 μL of each properly diluted sample was added in Falcon tubes to 5.850 mL of the DPPH* solution. The mixture was kept at rest for 15 min in the dark.

Absorbance readings were performed using a spectrophotometer (Femto Cirrus 80 ST, Femto, São Paulo, SP, Brazil) at a wavelength of 515 nm, with ethanol as the blank. The analyses were performed in sextuplicate. The calibration curve was constructed with Trolox (2 μmol L^−1^). The results were expressed as μM of Trolox equivalents per gram of sample (μM TE g^−1^ of sample), and the antioxidant activity was calculated using Equation (2).

(2) $$DPPH\;(µM\;TE\;g^{-1}\;of\;sample)=\frac{\left[X\right]Trolox\left(µmol\right)\times Total\;extract\;volume\;(mL)}{Sample\;mass\;(g)}$$

### 2.7. pH and Total Titratable Acidity (TTA)

For pH determination, a solution was prepared by macerating 5 g of black pepper with 50 mL of deionized water. The mixture was left to stand for 30 min at room temperature, and after decantation, the pH was measured using a portable digital pH meter (model K39-0014PA, Kasvi, São José dos Pinhais, PR, Brazil).

The total titratable acidity (TTA), expressed as a percentage, was determined via titration using a 0.1 mol/L sodium hydroxide (NaOH) solution standardized with potassium biphthalate, following the standards of the Instituto Adolfo Lutz [41]. For the analysis, 50 mL of distilled water were added to 5 g of whole black peppercorns. The titration was then carried out with 0.1 mol/L NaOH, in triplicate, until the solution reached pH 8.2, measured with a portable digital pH meter (model K39-0014PA, Kasvi, São José dos Pinhais, PR, Brazil). The volume of NaOH used in the titration was applied to calculate the TTA (Equation (3)), with results expressed as citric acid equivalents (%, g 100 g^−1^).

(3) $$Citric\;acid\;(\%,g\;100\;g^{-1})=\frac{V\times f\times 10}{m}$$

where:

V—volume of sodium hydroxide used in the titration (mL)

F—correction factor

M—sample mass (g)

### 2.8. Color

Color measurements of the samples were performed using a colorimeter (model CR–410, Konica Minolta, Osaka, Japan). Color evaluation was based on the reflectance of the CIELab coordinates (Commission Internationale de l’Eclairage): L* (lightness from white to black), a* (red to green), and b* (yellow to blue).

The samples were placed in transparent plastic bags, and color was measured at four random points of each sample. Based on the L*, a*, and b* values, the color difference (∆E*) and color saturation or chroma (C*) were determined using Equations (4) and (5), respectively.

(4) $$∆E^{*}=\sqrt{\left({\left(∆L^{*}\right)}^{2}+{\left(∆a^{*}\right)}^{2}+{\left(∆b^{*}\right)}^{2}\right)}$$

(5) $$C^{*}\;=\sqrt{\left({\left(a^{*}\right)}^{2}+{\left(b^{*}\right)}^{2}\right)}$$

### 2.9. Moisture Content

The moisture content of black peppercorns was determined using the gravimetric method, in which the samples were subjected to 105 ± 3 °C for 24 h, as described by the Association of Official Analytical Chemists [42]. The result was expressed on a wet basis (% w.b.).

### 2.10. Extraction of Black Peppercorn Essential Oil

The essential oil from black peppercorns was extracted via hydrodistillation. Initially, the black peppercorns exposed to ozone gas and not inoculated with *Salmonella* were ground in a blender (model OLIQ601, Oster, São Paulo, SP, Brazil) for 2 min. After grinding, the material was sieved using a 1.0 mm mesh sieve. Then, 60 g of black peppercorns and 500 mL of distilled water were transferred to a 1000 mL round-bottom flask connected to a Clevenger apparatus and heated using a heating mantle (Warmnest, São Paulo, SP, Brazil). Three replicates were performed for each treatment. To improve the efficiency of essential oil extraction, an ultrasonic pre-treatment (20 min) was applied to all black pepper samples (model Q335D, Quimis, Diadema, SP, Brazil).

The extraction time was recorded from the moment the first drop of essential oil was condensed and collected in the extraction system. After extraction, the black peppercorn essential oil was collected and stored at 4 °C. The extraction yield was calculated using Equation (6).

(6) $$Yield\;of\;black\;peppercorn\;EO\;(\%)=\left(\frac{Amount\;of\;EO\;obtained\;(g)}{Amount\;of\;black\;peppercorns\;(g)}\right)\times 100$$

where: EO—essential oil

#### 2.10.1. Identification of Major Compounds in Black Pepper Essential Oil

The identification of major compounds was performed via gas chromatography coupled with mass spectrometry (GC-MS), using a GCMS-QP2010 chromatograph (Shimadzu, Kyoto, Japan) equipped with an automatic injection system (AOC-20i, Shimadzu, Kyoto, Japan).

The optimized analytical conditions for chromatographic separation included a capillary column SH-Rtx-5MS (30 m × 0.25 mm × 0.25 µm) with a stationary phase composed of 5% phenyl and 95% dimethylpolysiloxane, using helium (99.999% purity, White Martins, Rio de Janeiro, RJ, Brazil) as the carrier gas, at a flow rate of 1.17 mL min^−1^.

The initial column temperature was set at 50 °C, increased at a rate of 3 °C min^−1^ up to 150 °C, and held for 10 min. Then, the temperature was increased at 20 °C min^−1^ until reaching a maximum of 260 °C, which was maintained for 10 min. The total run time was 59.83 min. The injector, detector interface, and ion source temperatures were set at 220 °C, 300 °C, and 230 °C, respectively. A 1 µL volume of the sample diluted in ethanol was injected using a split ratio of 1:100.

The mass spectrometer was operated in SCAN mode, with mass spectra acquired using electron impact ionization at 70 eV and scanning from 35 to 500 *m*/*z*. Compound identification was performed by comparing the retention indices (RI), obtained by injecting a series of hydrocarbon standards (C7–C40) with those in the instrument’s database (NIST-14 library) and with literature data [43]. The relative percentage of each compound was calculated based on the ratio between the peak area of each compound and the total area of all constituents in the sample.

#### 2.10.2. Quantification of the Major Compounds of Black Pepper Essential Oil

The quantification of the major compounds in black pepper essential oil was performed using a gas chromatograph (model GC-2014, Shimadzu, Kyoto, Japan) equipped with a flame ionization detector (GC-FID). A capillary column (30 m × 0.25 mm × 0.25 μm) (DB-5, Shimadzu, Japan) was used, with nitrogen (99.999%, Air Products, São Paulo, SP, Brazil) as the carrier gas, at a flow rate of 1.3 mL min^−1^. The initial oven temperature was 45 °C (2 min), followed by a heating rate of 30 °C min^−1^ up to 260 °C. The injector and detector temperatures were set at 240 °C and 280 °C, respectively. The split ratio was 1:50, and the total analysis time was 13.83 min.

For the optimization process, essential oil samples were diluted 1000-fold in ethanol. The compounds were identified by comparing the retention times of the sample extract peaks with those of analytical standards. The concentrations of β-pinene and limonene were determined using calibration curves constructed from standard solutions of these compounds at concentrations ranging from 10 to 200 mg L^−1^.

### 2.11. Statistical Analyses

Data from microbiological analyses were evaluated using descriptive statistics. Data on bioactive compounds (color, pH, TTA, and moisture content), essential oil yield, and composition were subjected to analysis of variance (ANOVA) and regression analysis. Regression models were selected based on the significance of the regression coefficients, using Student’s *t*-test and the coefficient of determination (r^2^ or R^2^). Statistical analyses were performed using SAEG software, version 9.1 (2007, UFV, Viçosa, MG, Brazil).

## 3. Results

### 3.1. Ozonation in Flow

#### 3.1.1. Microbiological Analyses

The effect of ozone gas exposure in flow on the decontamination of black peppercorns is presented in Table 1. When applied in flow, ozone gas humidified with sodium chloride solution reduced *Salmonella* contamination at different exposure times, achieving total decontamination. At 2 and 4 h of exposure, the quantitative test showed that the pathogen population was below the detection limit (<1.0 CFU g^−1^), while the qualitative test indicated the presence of *Salmonella* in 25 g of the sample. Pathogen reduction increased with longer ozone gas exposure time, and the absence of *Salmonella* was observed in 25 g of black peppercorns exposed to ozone in flow for 8 h.

#### 3.1.2. TPC, Antioxidant Activity Using the DPPH Method, and Quality Analyses

Table 2 presents the fitted regression equations and determination coefficients (R^2^) describing the TPC, antioxidant activity (DPPH*, expressed as μM Trolox), moisture content (%), pH, TTA (%), and color (∆E*, L*, and C*) for treatments with ozone gas applied in flow. Variables that did not show significant effects were described using the overall mean.

In the analysis of black pepper before treatment, the initial moisture content was approximately 10.0% (w.b.), the pH was 5.7, and the TTA was 0.16%. Regarding color, the initial values were: lightness (L*), 47.26; color difference (∆E*), 1.98; and color saturation (C*), 2.23.

For the ozone treatments, a linear effect was observed for TPC, pH, and TTA. After 8 h of ozonation, the values estimated from the regression equations for TPC, pH, and TTA were 922.85 mg of gallic acid per 100 g, 6.18, and 0.24%, respectively. Antioxidant activity, L*, and C* followed a square root function. With each hour of ozone exposure, there was an average reduction of 25.475 mg of gallic acid per 100 g of sample in TPC. For antioxidant activity, a peak value of 45.8853 μM Trolox was observed when the ozonation time reached the critical point of 9 h 55 min. For the color parameters L* and C*, values of 46.4745 and 1.2836 were observed at the critical points of 52.26 min and 4 h 58 min of ozonation, respectively. However, ∆E* did not show significant variation after ozone treatment, with an average of 2.06.

Ozone treatment increased the pH of black peppercorns by 0.0695 per hour of ozonation. Similarly, the TTA increased with exposure time. Table 3 shows the Pearson correlation matrix between the quality parameters of black pepper. No significant correlations were observed among the studied quality parameters, except for pH and TTA, which showed a negative correlation.

### 3.2. Ozone Gas Application in a Closed Low-Pressure System

#### 3.2.1. Microbiological Analyses

Table 4 shows the results of the effect of ozone on packaged black peppercorns, applied under low pressure in a hypobaric chamber. A reduction greater than three log cycles of *Salmonella* is estimated when the number of injections reaches 10. With the increase to 15 and 20 injections, the counts were below the detection limit (<1.0 CFU g^−1^). In the control treatment, the *Salmonella* population was 10^3^ CFU g^−1^ (Table 1). In the qualitative test, the presence of *Salmonella* was observed in 25 g of the samples subjected to all numbers of ozone injections studied.

#### 3.2.2. TPC, Antioxidant Activity Using the DPPH* Method, and Quality Analyses

The adjusted regression equations and the respective determination coefficients (r^2^/R^2^) describing the behavior of TPC (mg of gallic acid 100 g^−1^ of the sample), antioxidant activity (DPPH*, expressed as μM TE g^−1^), moisture content (%), pH, TTA (%), and color (∆E*, L*, and C*) of black peppercorns subjected to low-pressure ozonation are shown in Table 5. A linear effect was observed for antioxidant activity (DPPH*), a quadratic effect for TTA, ∆E*, and C*, and a square root effect for the other variables.

The low-pressure ozone injection system altered the bioactive compounds in black peppercorns. The equation ${\hat{y}}_{i}$ = 1119.69 − 97.8567 * ni^1/2^ + 8.2652° estimated that the critical point would be reached with 35 injections, yielding a concentration of 589.3262 mg of gallic acid per 100 g^−1^ of the sample for TPC. Furthermore, antioxidant activity showed an average increase of 0.1259 μM of Trolox g^−1^ of the sample as the number of ozone injections in the hypobaric chamber increased. After 20 injections, a value of 58.09 μM TE g^−1^ was estimated based on the fitted regression equation.

The minimum estimated values for moisture content, pH, and TTA were 14.2765%, 4.7565, and 0.2456%, respectively, reached with 30, 10, and 11 ozone injections. For color, the values for L*, ∆E*, and C* were 46.0474, 2.4079, and 1.4201, respectively, at the minimum points of 2, 8, and 0.002 injections. Table 6 shows a positive correlation between antioxidant activity and moisture content (r = 0.906), as well as between moisture content and TTA (r = 0.708). On the other hand, negative correlations were observed between antioxidant activity and pH (r = −0.753), moisture content and pH (r = −0.917), and pH and TTA (r = −0.842). The other variables evaluated did not show statistically significant correlations.

### 3.3. Essential Oil Yield and Composition of Black Pepper Ozonized in Flow and at Low Pressure

The behaviors observed for essential oil yield and the quantification of the major compounds, β-pinene and limonene, are described in Table 7, which presents the regression equations and coefficients of determination (R^2^). Variables that were not significantly affected by the number of ozone injections are presented as overall means.

After chromatographic analysis via GC-MS, eight components of black pepper essential oil were identified: α-pinene (4.18%), sabinene (7.49%), β-pinene (11.04%), limonene (14.37%), α-copaene (5.4%), β-caryophyllene (53.37%), α-humulene (2.43%), and ϒ-bisabolene (1.73%). Due to their significant biological activities and the availability of certified analytical standards in the laboratory, the major compounds β-pinene and limonene were selected as representative components to be quantified via gas chromatography. The calibration curve for the quantification of β-pinene and limonene present in the essential oils was constructed using concentrations of both compounds ranging from 10 to 200 mg L^−1^.

The results show that the essential oil yield of black pepper ozonized in flow and at low pressure was not significantly influenced by ozone treatment (*p* > 0.05), when compared to non-ozonized samples (control). This suggests that ozonation times (2, 4, and 8 h) and the number of injections (10, 15, and 20) did not affect the extraction yield of the essential oil. The average essential oil extraction yield obtained was 0.74%. Likewise, the concentrations of β-pinene and limonene did not show significant changes (*p* > 0.05) when the peppercorns were treated under flow conditions. On the other hand, treatment of black pepper in the hypobaric chamber positively altered the chemical composition of β-pinene and limonene. The average concentrations observed for β-pinene and limonene were 117.99 and 90.34 mg·L^−1^, respectively. Although essential oils are composed of a complex mixture of compounds, β-pinene and limonene were selected for quantification due to their predominance in the composition and relevance to black pepper’s sensory and functional properties.

## 4. Discussion

### 4.1. Flow Ozonation

#### 4.1.1. Microbiological Analyses

The effectiveness of ozone in flow for 8 h in decontaminating black peppercorns contaminated with *Salmonella* may be associated with the exposure time and the use of a sodium chloride solution for gas humidification during the ozonation process. The presence of the solution prevented the grains from drying out, maintaining a moisture content of 10.01% during treatment, without compromising the ideal storage level, making *Salmonella* more susceptible to the treatment [44]. Additionally, the sodium chloride solution may have improved the solubility and increased the reactivity of the ozone, facilitating its penetration and causing membrane rupture, leading to bacterial death.

Studies indicate that microbial inactivation using ozone occurs through two main mechanisms: the oxidation of sulfhydryl groups of amino acids present in enzymes and the oxidation of polyunsaturated fatty acids in the cell wall [45,46]. Besides these mechanisms, other studies suggest that ozone may influence the overall polarity of the bacterial surface [47] and trigger lipid peroxidation mechanisms [48].

The ozone gas generation process via dielectric barrier discharge requires an air source free of moisture and rich in oxygen. As a result of electrical discharges, the temperature of the gas increases, which may contribute to ozone gas having a drying potential [49]. Therefore, humidifying the ozone gas with a sodium chloride solution allows for controlling the relative humidity during its application, preventing water loss from the grains and ensuring greater efficiency in microbial inactivation.

Previous studies have shown that ozone gas applied to products with higher moisture content has greater efficiency in microbial decontamination [50,51,52]. Zhao and Cranston [50] observed a reduction of 3 log cycles in black peppercorns that were previously moistened before the ozone treatment. These authors reported that the higher the moisture content of the grains, the greater the reduction of *Salmonella*. Therefore, increasing the moisture content of the grains, relative humidity, and treatment temperature tends to enhance the antimicrobial action of ozone gas [53]. This is because there is a positive correlation between the increased relative humidity and the toxicity of ozone gas [54].

The reduction of about 99.9 to 99.99% in the number of *Salmonella* inoculated on black pepper observed with the treatment of sodium chloride humidified ozone gas in flow for 2, 4, and 8 h demonstrates the effectiveness of the method for disinfecting this grain contaminated with this pathogen. Similar reduction values have been observed with other decontamination methods. Gabriel et al. [55] found a 99.86% reduction of *Salmonella enterica* in black peppercorns irradiated with UV-C. Song and Kang [56] reported a reduction of 4.52 log CFU g^−1^ (99.99%) when decontaminating black and red pepper contaminated with *Salmonella* Typhimurium using hydrogen peroxide vapor. Kim et al. [57] observed a reduction greater than 5 log (99.999%) when applying combined ultraviolet radiation from vacuum amalgam lamps and near-infrared radiation heating in the decontamination of black pepper contaminated with *Escherichia coli* O157:H7 and *Salmonella enterica* serovar Typhimurium. However, with the use of ozone, the reduction values in the *Salmonella* count were lower. A reduction of 1.57 and 1.66 log CFU/g (97%) was reported in red pepper treated with high-concentration short exposure ozone gas and low-concentration long exposure, respectively, on the pathogen population and aflatoxin B1 concentration [58]. On the other hand, Darra et al. [59] observed the absence of *Salmonella* in 25 g of black pepper treated with ozone applied in a fluidized bed.

#### 4.1.2. TPC, Antioxidant Activity Using the DPPH* Method, and Quality Analyses

Phenolic compounds possess antioxidant activity and exhibit anti-inflammatory, anticancer, antimicrobial, antitumor, and anti-ulcer properties [60]. Therefore, phenolic compounds in plants can play a significant role as antioxidants. Alothman et al. [61] reported a decrease in TPC in the control treatment of freshly cut tropical fruits, from an initial value of 178.51 ± 3.60 mg GAE 100 g^−1^ to 167.96 ± 0.12 mg GAE 100 g^−1^ after 10 min, 145.78 ± 0.12 mg GAE 100 g^−1^ after 20 min, and 96.51 ± 0.12 mg GAE 100 g^−1^ after 30 min of ozone exposure. On the other hand, Nagy et al. [62], while investigating the phenolic composition of various spices, including black pepper, found a TPC of 338 mg GAE 100 g^−1^ in untreated pepper. In contrast, Shelake et al. [63] observed an increase in TPC in onion bulbs from 57.71 ± 0.09 mg GAE 100 g^−1^ (control) to 59.98 ± 0.12 mg GAE 100 g^−1^ after treatment with gaseous ozone at 400 ppm. The same authors noted that exposure to 600 and 900 ppm resulted in phenolic degradation. This effect may be attributed to ozone’s ability to generate several free radicals, such as hydroperoxyl, hydroxyl, and superoxide radicals [64], which degrade phenolic compounds in primary products when applied at high concentrations [61].

Similar results to those of the present study were reported by Liu et al. [65], who found that exposing garlic to 5 ppm of ozone for 15 min induced an increase in TTA to 13.87% after 10 days of storage, compared to the control (12.38%). Acidity is a key parameter in food preservation, as spoilage processes such as hydrolysis, fermentation, or oxidation typically result in changes in hydrogen ion concentration [41].

Color loss in black pepper during the decontamination process is a critical factor for consumer acceptance [66]. Ozonation can lead to significant changes in the color of food products due to its strong oxidative properties. This process may degrade natural pigments, leading to a decrease in C*, an increase in L*, and reduced overall color stability. The extent of these changes depends on factors such as ozone concentration, exposure time, and the specific food matrix [67]. In our study, minimum values of 46.47 for L* and 1.28 for C* were observed at 52 min and 4 h 58 min of ozone exposure, respectively. Although significant variations were observed when analyzing the L* and C* variables, these changes can be considered not meaningful. On other hand, Wang et al. [68] observed an increase in the L* value from 93.64 ± 0.12 (control) to 94.25 ± 0.02 after 30 min of ozonation in wheat flour. Similarly, Sharma et al. [69], working with foxtail millet (*Setaria italica*) flour, reported an increase in the L* value with longer ozonation times. The lighter color observed in black pepper samples treated with ozone may be attributed to its bleaching effect due to its strong oxidizing potential. Conversely, Dogu-Baykut et al. [66] found no significant differences in L* values in ozone-treated black pepper.

In the present study, the moisture content of 10.01% in black peppercorns across all treatments was below the maximum limit established by Normative Instruction No. 10, dated May 15, 2006 [70], which sets 14% as the maximum acceptable moisture content for black pepper. This reduced moisture content hinders the growth of undesirable microorganisms, preventing contamination that could pose a risk to consumer health. Water activity directly influences microbial proliferation, thereby affecting both the quality and safety of black pepper [71].

### 4.2. Ozone Application in a Closed Low-Pressure System

#### 4.2.1. Microbiological Analyses

The injection of ozone under low pressure is a technique that has been studied for the treatment and microbiological decontamination of packaged products [30,72]. However, there are still no reports of studies evaluating its effectiveness in bacterial control when applied via injection in low-pressure systems for packaged products. The present study is the first to report the effects of ozone treatment in a hypobaric chamber for the decontamination of *Salmonella* in black pepper. It was observed that from 15 injections onward, there was a significant reduction in *Salmonella* counts in the quantitative test (Table 4). The pathogen population was below the detection limit using the pour plate technique. The low number of surviving cells may be related to the increased ozone concentration within the mass of peppercorns. However, despite the reductions observed in the quantitative test, the qualitative test indicated the presence of *Salmonella* in 25 g of sample even when 20 injections were applied (Table 4).

To achieve higher levels of efficiency and ensure the absence of *Salmonella* contamination in 25 g of the sample, it is suggested to use a greater number of injections and a further reduction of the internal pressure of the hypobaric chamber. In this study, the pressure was reduced to 350 hPa, whereas Silva et al. [30], when investigating the action of ozone injected under low pressure for the control of *Sitophilus zeamais* in popcorn, reduced the internal chamber pressure to 250 hPa. The greater the pressure gradient established between the ozone injection point and the interior of the hypobaric chamber, the higher the concentration levels that will be reached inside the packaging and, consequently, the greater the treatment efficiency.

The main advantage of low-pressure ozone injection treatment is the possibility of treating already-packaged products, thus avoiding additional handling. This strategy can be especially useful in distribution centers, where products are already prepared for commercialization. In this context, this application strategy requires further scientific investigation to improve the use of this technology for the microbiological decontamination of spices.

#### 4.2.2. TPC, Antioxidant Activity Using the DPPH* Method, and Quality Analyses

The observed increase in TPC and antioxidant activity, as measured via the DPPH* method, can be attributed to the ability of ozone gas to cleave covalent bonds within complex polymeric compounds, thereby releasing bound phenolic compounds and antioxidants [73]. This release potentially enhances the antioxidant capacity of the treated black peppercorns, which is significant because phenolics are well-known for their role in scavenging free radicals and protecting biological molecules from oxidative damage [74].

An increase in moisture content was observed, which can be explained by the fact that all the gas injected into the hypobaric chamber remained inside it, thus preventing moisture loss from the grains [30]. The absence of moisture loss has also been reported in other studies, such as those involving the treatment of common beans (*Phaseolus vulgaris*), cowpeas (*Vigna unguiculata* L.), corn (*Zea mays* L.), paddy rice (*Oryza sativa*), polished rice, and popcorn (*Zea mays everta*) treated with ozone applied under low pressure [30,72]. Moisture retention in treated grains can be beneficial for maintaining texture and reducing shrinkage but may also influence microbial stability and shelf-life, aspects that warrant further study.

Regarding color, increases in L* and C* values were observed with the growing number of ozone injections, suggesting that ozone treatment affects the visual attributes of the grains, potentially through oxidation or modification of pigment compounds on the grain surface. On the other hand, ∆E* values decreased with each ozone injection in the hypobaric chamber. This result contrasts with findings in the literature, which indicated no change in ∆E* for packaged common beans, cowpeas, corn, paddy rice, polished rice, and popcorn exposed to ozone gas [30,72]. This discrepancy might be due to differences in the grain matrix, ozone concentration, or exposure duration. Since color is a critical quality attribute that affects consumer acceptance, these changes should be carefully evaluated in future studies.

Future research directions could include more detailed chemical analyses to identify specific phenolic compounds released or modified by ozone treatment, investigations into how moisture retention affects storage stability and sensory properties, and the use of complementary antioxidant assays (e.g., ABTS, FRAP) to provide a more comprehensive assessment of antioxidant potential.

### 4.3. Extraction Yield and Composition of Essential Oil Constituents from Black Pepper Treated with Ozone in Flow and Low Pressure

The absence of significant changes in oil yield following ozonation may be explained by the limited impact of ozone on the structural integrity of the oil storage sites within black pepper fruits. Essential oils are typically stored in specialized secretory structures such as glandular cells or ducts, which are physically protected within the pericarp. The oxidative action of ozone, although effective at microbial decontamination on the surface, may not have been intense or prolonged enough to disrupt these internal structures. Although treatment of black pepper in a hypobaric chamber did not affect the essential oil extraction yield, it favored an increase in the concentrations of β-pinene and limonene.

One hypothesis is that ozone, acting as an oxidizing agent, may induce modifications in the polysaccharide matrix of the pericarp cell walls of black pepper, increasing the permeability of the oil-bearing structures without fully disrupting their oil-storage integrity. This would facilitate the selective release of certain monoterpenes (such as β-pinene and limonene), elevating their concentration in the extracted phase while leaving the total essential oil yield unchanged.

Ouf et al. [75] observed in their study an increase in the main biologically active constituent associated with the medicinal properties of chamomile flowers and oxidative degradation of low molecular weight compounds in peppermint after exposure to 3 ppm of ozone for 280 min. Furthermore, Zhao et al. [50] reported that ozone treatment of ground black pepper led to slight oxidation of the volatile constituents of the essential oil. Ozone influenced the volatile constituents of the extracted oil, altering approximately 8.33% for β-pinene and 3.05% for limonene.

In the present study, flow ozonation with humidification of the ozone gas using a sodium chloride solution proved effective, as it prevented moisture loss in black pepper and showed superior performance both in decontamination and in preserving the physicochemical attributes of the grains. On the other hand, the application of ozone under low pressure was not effective in inactivating *Salmonella* in black pepper grains.

## 5. Conclusions

The treatment of black pepper with ozone gas humidified with a sodium chloride solution in flow for 8 h proved to be the most effective application condition for the decontamination of black pepper contaminated with *Salmonella* and for the preservation of the physicochemical quality of the grains. Under the tested conditions, although the low-pressure injection system achieved reductions in bacterial counts, it did not reach the condition of *Salmonella*-free contamination in 25 g of the sample.

In addition to the antimicrobial effects, the treatment did not significantly influence the essential oil yield or the contents of its major constituents, β-pinene and limonene, particularly under flow conditions. However, changes in the chemical composition of these compounds were observed following ozone application under low pressure, indicating a potential effect of the oxidative environment on the essential oil composition depending on the treatment system.

This study highlights the potential of humidified flow ozonation as a non-thermal decontamination method. However, some limitations must be acknowledged. The low-pressure system, even with multiple injections, was not sufficient for total microbial inactivation, indicating the need for process optimization (e.g., increasing the exposure time or adjusting the pressure and humidity parameters). Additionally, only the DPPH* method was used to assess antioxidant activity, and a broader set of antioxidant assays could provide a more comprehensive understanding of the treatment’s impact. Future studies should also investigate the effects of ozone treatments on sensory attributes and long-term microbial stability to evaluate the commercial applicability of this method in the food industry.

## Figures and Tables

**Table 1:** Microbiological analysis of *Salmonella* (quantitative) and presence/absence of *Salmonella* (qualitative) in black peppercorns treated with ozone gas humidified with sodium chloride solution in flow (16 mg L^−1^), at different exposure times and control treatment (inoculated and not exposed to ozone gas, left under ambient conditions for 2, 4, and 8 h).

Treatments	*Salmonella* Determination	
	Quantitative (log CFU g^−1^) ^a^	Qualitative (Presence/Absence in 25 g)
**Control/0 h**	3.87 ± 0.26	**
**Control/2 h**	4.21 ± 0.06	**
**16 mg L^−1^/2 h**	˂1.0 *	Present
**Control/4 h**	3.13 ± 0.11	**
**16 mg L^−1^/4 h**	˂1.0 *	Present
**Control/8 h**	4.62 ± 0.07	**
**16 mg L^−1^/8 h**	˂1.0 *	Absent

^a^ Values represent the mean ± standard deviation. * Below the detection limit of the method. ** Qualitative analysis not performed. CFU: colony-forming unit.

**Table 2:** Fitted regression equations for total phenolic content (TPC), antioxidant activity (DPPH*), moisture content, pH, total titratable acidity (TTA), lightness (L*), color difference (∆E*), and color saturation (C*) in black peppercorns treated with ozone in flow, as a function of exposure time (et), and their respective determination coefficients (r^2^/R^2^).

Variables	Model Type	Equation	r^2^/R^2^
**TPC**	Linear	${\hat{y}}_{i}$ = 1126.65 − 25.475 * et	0.8916
DPPH*	Square root	${\hat{y}}_{i}$ = 43.7272 + 0.8146 * et^1/2^ − 0.1293 ° et	0.9952
**Moisture content**	-	${\hat{y}}_{i}$ = 10.01	-
**pH**	Linear	${\hat{y}}_{i}$ = 5.6266 + 0.0695 ** et	0.9697
**TTA**	Linear	${\hat{y}}_{i}$ = 0.1507 + 0.0115 ** et	0.9767
L*	Square root	${\hat{y}}_{i}$ = 47.26 − 1.8129 ** et^1/2^ + 0.9713 ** et	0.9998
∆E*	-	${\hat{y}}_{i}$ = 2.06	-
C*	Square root	${\hat{y}}_{i}$ = 2.2138 − 0.5379 **^▪^** te^1/2^ + 0.1206 **^□^** et	0.9212

** Significant at 1% probability using the *t*-test (*p* < 0.01); * Significant at 5% probability using the *t*-test (*p* < 0.05); ° Significant at 10% probability using the *t*-test (*p* < 0.10); **^▪^** Significant at 15% probability using the *t*-test (*p* < 0.15); ^□^ Significant at 20% probability using the *t*-test (*p* < 0.20).

**Table 3:** Pearson correlation matrix between the quality parameters of black peppercorns subjected to flow ozonation.

Variables	TPC ^1^	DPPH* ^2^	Moisture Content	pH	TTA	L*	∆E*	C*
**TPC**	1.000	−0.130	0.239	0.163	−0.204	−0.373	−0.021	−0.356
DPPH*		1.000	−0.307	−0.477	0.287	−0.024	0.198	−0.325
**Moisture content**			1.000	−0.412	0.484	0.279	0.386	−0.048
**pH**				1.000	−0.922 *	−0.260	−0.275	−0.117
**TTA**					1.000	0.251	0.316	0.042
***L*** *						1.000	0.036	0.313
∆E*							1.000	−0.388
***C*** *								1.000

* Significant at 5% probability (*p* < 0.05). ^1^ Expressed as mg of gallic acid per 100 g of sample. ^2^ Expressed as μM Trolox. TPC: total phenolic content ^1^; DPPH*: antioxidant activity ^2^, TTA: total titratable acidity; L*: lightness; ∆E*: color difference; C*: color saturation.

**Table 4:** Quantitative analysis of *Salmonella* and presence/absence of *Salmonella* (qualitative) in black peppercorns treated with ozone in a closed low-pressure system.

Treatments	*Salmonella* Determination	
	Quantitative (log CFU g^−1^) ^a^	Qualitative (Presence/Absence in 25 g)
**0 injections**	3.87 ± 0.26	-
**10 injections**	3.25 ± 0.104	-
**15 injections**	˂1.0 *	Present
**20 injections**	˂1.0 *	Present

^a^ The values represent the mean ± standard deviation. * Below the detection limit of the technique. CFU: colony-forming unit.

**Table 5:** Fitted regression equations for total phenolic content (TPC), antioxidant activity (DPPH*), moisture content, pH, titratable acidity (TTA), lightness (L*), color difference (∆E*), and color saturation (C*) in packaged black peppercorns treated with ozone in a sealed low-pressure system, as a function of the number of injections (ni), and their respective coefficients of determination (r^2^/R^2^).

Variables	Model	Equations	r^2^/R^2^
**TPC**	Square root	${\hat{y}}_{i}$ = 1119.69 − 97.8567 * ni^1/2^ + 8.2652 ° ni	0.9992
DPPH*	Linear	${\hat{y}}_{i}$ = 55.5732 + 0.1259 ** ni	0.9922
**Moisture content**	Square root	${\hat{y}}_{i}$ = 9.9903 + 0.8536 ** ni^1/2^ − 0.0773 * ni	0.9999
**pH**	Square root	${\hat{y}}_{i}$ = 5.6652 − 0.3431 ° ni^1/2^ + 0.0545 ° ni	0.9835
**TTA**	Quadratic	${\hat{y}}_{i}$ = 0.1565 + 0.0158 ** ni − 0.0007 ** ni^2^	0.9996
L*	Square root	${\hat{y}}_{i}$ = 47.2132 – 1.2412 ° ni^1/2^ + 0.4312 ° ni	0.5762
∆E*	Quadratic	${\hat{y}}_{i}$ = 1.9399 + 0.1185 ° ni − 0.0075 ° ni^2^	0.7379
C*	Quadratic	${\hat{y}}_{i}$ = 2.2280 − 0.0557 * ni + 0.0009 * ni^2^	0.9993

** Significant at 1% probability using the *t*-test (*p* < 0.01); * Significant at 5% probability using the *t*-test (*p* < 0.05); ° Significant at 10% probability using the *t*-test (*p* < 0.10).

**Table 6:** Pearson correlation matrix between the quality parameters of black peppercorns treated with low-pressure ozonation.

Variables	TPC ^1^	DPPH* ^2^	Moisture Content	pH	TTA	L*	∆E*	C*
**TPC**	1.000	0.182	0.304	−0.135	0.271	−0.330	0.224	−0.491
DPPH*		1.000	0.906 *	−0.753 *	0.479	0.457	−0.029	−0.031
**Moisture content**			1.000	−0.917 *	0.708 *	0.353	−0.100	0.002
**pH**				1.000	−0.842 *	−0.236	0.040	−0.126
**TTA**					1.000	0.174	0.081	0.038
***L*** *						1.000	−0.370	0.384
∆E*							1.000	−0.353
***C*** *								1.000

* Significant at 5% probability (*p* < 0.05) ^1^ Expressed as mg of gallic acid per 100 g of sample. ^2^ Expressed as μM Trolox. TPC: total phenolic content ^1^; DPPH*: antioxidant activity ^2^; TTA: total titratable acidity; L*: lightness; ∆E*: color difference; C*: color saturation.

**Table 7:** Adjusted equations for extraction yield, β-pinene, and limonene content of essential oil from black pepper ozonized in flow and at low pressure, as a function of exposure time (et) or number of injections (ni), and the respective coefficients of determination (R^2^).

Variables	Model	Equations	R^2^
**Flow**			
Yield	-	${\hat{y}}_{i}$ = 0.7368	-
β-pinene	-	${\hat{y}}_{i}$ = 117.99	-
Limonene	-	${\hat{y}}_{i}$ = 90.34	-
**Hypobaric chamber**			
Yield	-	${\hat{y}}_{i}$ = 0.7868	-
β-pinene	Square root	${\hat{y}}_{i}$ = 131.08 − 29.2906 ° ni^1/2^ + 4.9403 ° ni	0.9058
Limonene	Quadratic	${\hat{y}}_{i}$ = 97.9042 − 5.587 * ni + 0.2651 * ni^2^	0.9887

* Significant at 5% probability using the *t*-test (*p* < 0.05); ° Significant at 10% probability using the *t*-test (*p* < 0.10).

## Data Availability

The original contributions presented in the study are included in the article, further inquiries can be directed to the corresponding author.
